# Epilepsy

**Published:** 1846-02

**Authors:** 


*Epilepsy.*—Dr. Macdonald reported a case of epilepsy in a man aged 45 years, who previously had enjoyed fair health; although he had had symptoms of dyspepsia. He was attacked during the night, after returning from a fishing excursion, having been observed by a friend who slept in the same room. Dr. M. saw him in the morning; he continued in the epileptic state till 12, A.M., without five minutes' cessation. The convulsions were frightful; and so severe that it was ten days before he became conscious of his situation. The pulse remained unaltered. He was bled, and counter-irritants were used all over the body. Administered 220 drops of tincture of opium in two separate portions. Convulsions shortly ceased.—*Ibid.*

